# Myths of ADHD in the Kyrgyz Republic: Destigmatization and Challenges in Mental Health

**DOI:** 10.1192/j.eurpsy.2025.1489

**Published:** 2025-08-26

**Authors:** E. Molchanova

**Affiliations:** 1 Psychology, American University in Central Asia, Bishkek, Kyrgyzstan

## Abstract

**Introduction:**

As a neurodevelopmental disorder characterized by symptoms such as inattention, hyperactivity, and impulsivity, ADHD was once predominantly associated with Western medical discourse. However, recent years have seen a notable rise in self-diagnoses of ADHD among young people in Kyrgyzstan.

**Objectives:**

This research aims to explore the myths surrounding ADHD in the Kyrgyz Republic, focusing on how these misconceptions contribute to the destigmatization of mental health disorders and create challenges for mental health professionals. The research also seeks to examine the gap between public awareness of ADHD and the actual resources available for diagnosis and treatment in Kyrgyzstan.

**Methods:**

This analysis is qualitative and is based on, (1) observations of self-diagnosis trends among young people in Kyrgyzstan, and (2) anecdotal evidence from mental health professionals working in the country. In-depth interviews were conducted with 15 adolescents, self-diagnosed with ADHD, and 15 mental health care specialists working in Bishkek. Informed consent was presented before the interviews and both patients and specialists were rewarded by 15 dollars after the interview.

**Results:**

Three key myths emerged from the analysis: (1) **ADHD as a Temporary Condition:** A prevalent belief in Kyrgyzstan is that ADHD can be easily cured by a few visits to a “good” psychologist. It reflects the unrealistic expectations of therapy and the lack of understanding that ADHD often requires long-term management. (2)**ADHD as a Fashionable Disorder:** There is a growing trend, particularly among urban youth, to romanticize ADHD as a “cool” or “fashionable” diagnosis. While this has contributed to greater awareness of ADHD, it also trivializes the disorder, promoting self-diagnosis and diluting the seriousness of the condition. (3) **ADHD as a Childhood Disorder:** Another widespread misconception is that ADHD primarily affects children and that individuals grow out of it with age. This myth prevents many from seeking early intervention.

**Conclusions:**

The growing awareness of ADHD in the Kyrgyz Republic is a double-edged sword. On one side, it is helping to reduce the stigma associated with mental health disorders, providing people with a socially acceptable way to discuss and understand their struggles. Conversely, the myths accompanying this awareness hinder effective diagnosis and treatment, making it difficult for individuals to access proper care. Mental health professionals must not only treat ADHD but also work to correct these societal misconceptions, a task made more difficult by limited resources in Kyrgyzstan. A more nuanced public understanding of ADHD, supported by accurate information and improved mental health infrastructure, is essential for addressing these challenges

**Disclosure of Interest:**

None Declared

